# Intrathoracic migration of a Steinman wire: a case report and review of the literature

**DOI:** 10.4076/1757-1626-2-8321

**Published:** 2009-07-14

**Authors:** Neoptolemos N Sergides, Dimitrios D Nikolopoulos, Dimitrios K Yfadopoulos, Evanthia A Novi, Maria P Kanata

**Affiliations:** 1 Orthopaedic Department, Central Clinic of Athens - Diagnostic and Treatment CenterAsklepiou 31 str, 106-80Greece; 2 Surgical Department of Thorax and Vascular Surgery, Central Clinic of Athens - Diagnostic and Treatment CenterAsklepiou 31 str, 106-80Greece; 3 Central Clinic of Athens - Diagnostic and Treatment CenterAsklepiou 31 str, 106-80Greece

## Abstract

**Introduction:**

Migration of orthopaedic fixation wires into the thoracic cavity occurs infrequently, but can have dire consequences. Although rare, intrathoracic migration is a serious complication that demands immediate removal.

**Case presentation:**

We present a case of a 59-year-old man with an intrathoracic migration of a Steinman wire used for the treatment of a shoulder fracture. Surprisingly, the migration was asymptomatic. The Steinman wire was successfully retrieved from the thorax via thoracotomy.

**Conclusion:**

The migration of pins and wires can cause fatal complications and should be considered as very hazardous. Therefore, if wires need to be used, terminally threaded pins are safer and the free end should be bent. The patients should be frequently followed, both clinically and radiographically, until all the wires are removed.

## Introduction

Pins and wires are used extensively for internal fixation of fractures in orthopaedic surgery, especially in the management of fractures and dislocations of the shoulder. Some of these internal fixation devices (Steinman, Kirschner wires) have a tendency to migrate. Although uncommon, when the migration of these wires occurs, there could be devastating consequences. Thus, prompt recognition and immediate retrieval of the implant is paramount to avert those complications [1,2]. In this study, we present the case of a 59-year-old man in whom a threaded Steinman, previously placed into the right shoulder, fixing a fracture, migrated into the right lung parenchyma; and was successfully retrieved via thoracotomy.

## Case presentation

**Figure 1.**
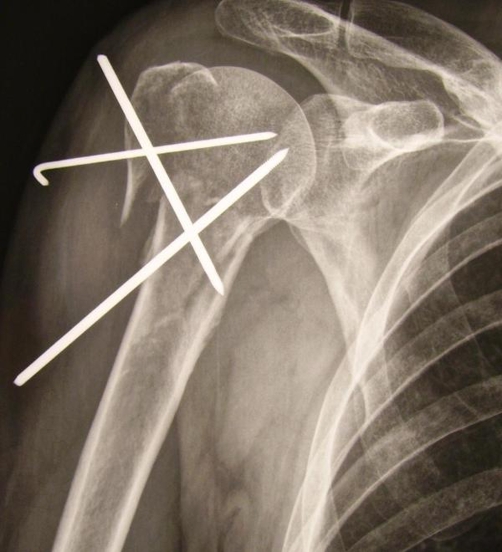
Post-operative radiograph of the three-part fracture of the right humerus head be fixed with three percutaneous Steinman wires.

**Figure 2.**
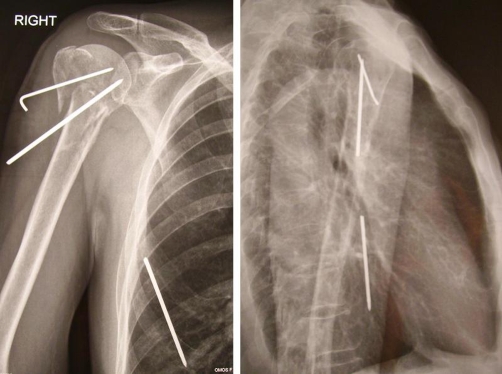
Intrathoracic migration of a Steinman wire; post-operative radiograph on the third week.

**Figure 3.**
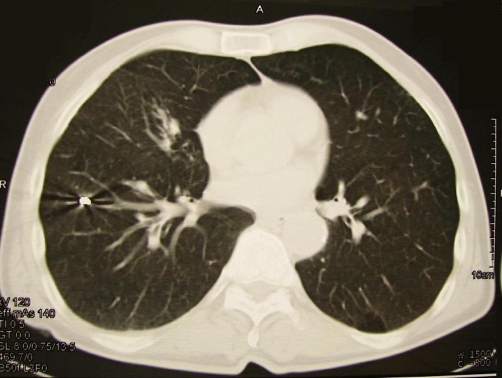
A chest CT-scan that performed, presented the Steinman wire to be located in the superior segment of the right lower lobe.

In September 2007, a 59-year-old Caucasian, drug addicted man was admitted into the orthopaedic department of our clinic after a low-velocity, three-part fracture of the right humerus head (Neer type IV - surgical neck and greater tuberosity fragment). He had a medical history of HBV, HCV and HIV positive, Brody myopathy and excised eosinophil granule of the 3^rd^ and 4^th^ right ribs. After a closed reduction, three percutaneous Steinman wires (threaded) were used for the fixation of the fracture (Figure 1). The patient was discharged the second postoperative day. On the third follow-up, three weeks postoperative, radiography was performed and surprisingly one Steinman was noted to be missing from the right shoulder. A chest radiograph confirmed its migration into the right hemithorax (Figure 2). The patient had no cardiopulmonary symptoms, and there was no evidence of a pneumothorax or a hemothorax. A chest CT-scan was performed (Figure 3) and the wire was located in the superior segment of the right lower lobe, but did not enter the mediastinum or the pleural space.

Because of the central position of the pin, prompt removal was recommended. The patient was scheduled for surgery the following day. He was placed in a left lateral decubitus position and a posterolateral thoracotomy in the 5^th^ right intercostal space was used. The Steinman wire, be lodged in the parenchyma of the right lower lobe, was grasped and slowly withdrawn from the lung. Once removed, the lung was re-expanded without difficulty. As a precaution, a 28F chest tube was placed under direct vision. There was no air leak or bleeding postoperatively and the tube was removed on the second postoperative day. The patient was discharged the following day. Four weeks later the other two Steinman wires on the right humeral head were removed under local anaesthesia.

## Discussion

Orthopaedic procedures frequently involve utilization of internal fixation devices (Steinman, Kirschner wires and pins). Migration of these devices within the chest is uncommon, but is a known complication, particularly with fixation wires or pins around the shoulder. Mazet reported in 1943 the first two cases of migration of a Kirschner wire from the shoulder region [1]. In 1990, Lyons and Rockwood published a review of all the reported cases of pin migration in operations on the shoulder [2]. It is noteworthy that, in 17 out of 47 cases the wire migrated to a major vascular structure, and in 21 out of 47 the wire had been used for internal fixation of a dislocated sternoclavicular joint [2].

Migrations usually follow a retrograde path and the wires protrude near the entry point. Occasionally migration occurs in antegrade direction, as in our case [3]. A recent review of the literature showed sporadic case reports of migrated pins and wires from the shoulder region to the spinal canal, the trachea, the spleen, the pulmonary artery, the heart, the mediastinum, the lung, the subclavian artery and the ascending and abdominal aorta [4-6]. Migration of K-wires has been reported as early as the first day and as late as 21 years [7] after the fixation. Usually the process causes no symptoms, but there have been observed deaths attributable to migration. All deaths were associated with catastrophic cardiovascular events and cardiac tamponade. Serious, non-fatal complications include pericardial tamponade, arrhythmia, pericarditis, pseudo-aneurysm, aortopulmonary fistula, pneumothorax, haemoptysis, subclavian steal syndrome, hemianopia, hemiplegia, paraplegia, radicular pain, dysphagia and splenic hematoma [4,6].

Various theories have been proposed to explain this migration, including muscular activity, regional resorption of bone, and the great freedom of motion of the shoulder. The latter may be responsible for the breakage of pins and wires that have been reported. The migration to the thorax seems to be favored by respiratory excursions, negative intrathoracic pressure, and gravitational forces [4,8]. This severe complication can be theoretical reduced by using threaded pins in surgical operations of the shoulder, by bending the free end of the wire, and lastly by removing the hardware as soon as possible [2,3]. Notwithstanding, in practice all these does not fully, one hundred percent, protect the patient against pin migration.

However, as evident in Chang et al. case, fracture and migration of a fractured fragment can still occur even if the outer end of the pin is bent and well-fixed [8]. Therefore, regular radiographic follow-up is needed for patients with internal fixation devices; any fractured pins or wires must be removed immediately to prevent dangerous migration. In other words, patients should be frequently followed both clinically and radiographically [4]. CT scan clearly evaluates the exact location of the migrated pin or wire, as well as existing complications.

Once migration of a pin or K-wire or Steinman is recognized, immediate surgical removal should be carried out. Sternotomy, thoracotomy and video-assisted thoracoscopy have been used [8]. Thoracoscopic removal of intrathoracic foreign bodies can be accomplished safely if the object can be withdrawn through a port site, if it does not traverse the mediastinum, and if the patient can tolerate single-lung ventilation [9]. Otherwise, thoracotomy and sternotomy are safe and could be used to remove migrated wires. Sternotomy may be considered more appropriate in the presence of injury to the heart or great vessels [5,10].

## Conclusion

Consequently, although our patient underwent a successful removal of the wires and recovered uneventfully, the migration of pins and wires can cause fatal complications and should be considered as very hazardous. Therefore, if wires need to be used, terminally threaded pins are safer and the free end should be bent. The patients should be frequently followed, both clinically and radiographically, until all the wires are removed.
